# Assessing emotion regulation difficulties in adolescents: validation and clinical utility of the difficulties in emotion regulation scale, 16-item

**DOI:** 10.1186/s40359-025-02540-3

**Published:** 2025-03-12

**Authors:** Kristina Holmqvist Larsson, Erik Aspeqvist, Fredrik Falkenström, Gerhard Andersson, Carl Göran Svedin, Maria Zetterqvist

**Affiliations:** 1 Department of Child and Adolescent Psychiatry in Linköping, Region Östergötland, Linköping, Sweden; 2 Center for Social and Affective Neuroscience, Department of Biomedical and Clinical Sciences, Linköping University, Linköping, Sweden; 3 Department of Psychology, Linnaeus University, Växjö, Sweden; 4 Department of Behavioural Sciences and Learning, Department of Biomedical and Clinical Sciences, Linköping University, Linköping, Sweden; 5 Department of Clinical Neuroscience, Karolinska Institute, Stockholm, Sweden; 6 Department of Social Sciences, Marie Cederschiöld University, Stockholm, Sweden

**Keywords:** Emotion regulation, DERS-16, Adolescents, Cluster analysis, Factor analysis

## Abstract

**Background:**

Emotion regulation difficulties have been identified as an underlying mechanism in the development and maintenance of psychopathology. The need to improve our understanding of emotion regulation difficulties to accurately assess and treat adolescents in child and adolescent psychiatric settings is essential.

**Method:**

In the first part of the study, the psychometric qualities of the Difficulties in Emotion Regulation Scale, 16-item version (DERS-16) were examined in a clinical child and adolescent psychiatric (CAP) sample. In the second part, the DERS-16 was used to examine emotion regulation difficulties in the CAP sample (*N* = 281, 15–19-year-olds, 77.6% female) and in a community sample of adolescents (*N* = 3,169, 16–19-year-olds, 55.6% female). Subgroups were further explored in the CAP sample by two-step cluster analysis with log-likelihood distance measures.

**Results:**

DERS-16 showed satisfactory psychometric qualities in the CAP sample. DERS-16 successfully distinguished adolescents in the clinical sample from adolescents in the community sample. Results showed significantly higher levels of self-reported emotion regulation difficulties in the CAP sample and in females. The two-step cluster analysis resulted in three clusters, named Minor, Moderate and Severe emotion regulation difficulties. Adolescents with the highest levels of emotion regulation difficulties had significantly more risk behaviors such as nonsuicidal self-injury and drug use, depression and anxiety, exposure to abuse, and higher levels of comorbidity.

**Conclusions:**

DERS-16 successfully distinguished clinical from community adolescents. The results illustrate the importance of identifying adolescents with high levels of emotion regulation difficulties in child and adolescent psychiatry due to higher levels of comorbidity and risk behaviors.

## Introduction

Emotion regulation (ER) is commonly explained as a mechanism to influence the intensity, duration, and expression of emotions [1–3]. To regulate emotions, we use strategies (functional or dysfunctional) in relationship to specific goals [4–6]. Difficulties in emotion regulation imply lack of strategies, or dysfunctional strategies [6], and is a transdiagnostic construct that is associated with several psychiatric conditions.

## Defining and assessing ER difficulties

Based on the relationship between ER, psychopathology, and risk behaviors in adolescents as well as in adults, it is important to detect and assess difficulties in ER in a reliable and valid manner [7, 8]. The research field of ER has grown considerably during the last decades and is still emerging. One of the challenges when measuring ER strategies, or lack of strategies, is that there is no agreed-upon definition of ER [9]. This lack of consensus contributes to the fact that there currently exist several measures that measure different types of ER. Adrian et al. [10] showed that a wide range of measures for measuring ER in children and adolescents were used in studies from 1975 to 2010, and since then even more measures have been developed. In addition to the lack of definitional agreement, there has also been some overlap with resembling concepts, such as coping [11], which further contributes to the range of measures.

Most definitions of ER emphasize the strategies that humans use to influence the intensity, duration, and expression of emotions [4, 5], focusing on modulating the experience of emotions rather than eliminating emotions [6]. Gratz and Roemer’s [6] conceptualization of ER; (a) awareness and understanding of emotions, (b) acceptance of emotions, (c) ability to control impulsive behaviors and behave in a goal directed way in the presence of negative emotions, and (d) ability to use situationally appropriate emotion regulation strategies, is widely used in ER research. Based on this conceptualization and definition of ER, Gratz and Roemer [6] developed the Difficulties in Emotion Regulation Scale (DERS). DERS contains 36 items and measures difficulties in ER in the four dimensions described above. In their original study of DERS, factor analysis resulted in a six-factor solution converted into the subscales: Nonacceptance, Impulse, Goals, Awareness, Strategy and Clarity which implies that the conceptualization of DERS is based on captures six rather than four dimensions of ER [6]. Other studies have suggested a five-factor model as best fit where the subscale Awareness is excluded [12, 13]. Bjureberg et al. [14] developed a short form of DERS, DERS-16, to increase clinical and research utility, especially in samples such as adolescents, where the 36-item version potentially could be too challenging. DERS-16 contains 16 items from the original scale with five out of the six original subscales. In the development of DERS-16, items were partly excluded based on item-total correlations less than *r* =.50, and none of the items from the lack of emotional awareness subscale was retained [14]. A bifactor model with five factors has shown acceptable to excellent fit in an adolescent sample with eating disorders [12], in adolescents with psychosis [15] and in adolescents with severe mental illness [16]. DERS-16 has shown excellent internal consistency with Cronbach’s α ranging from 0.92 to 0.95, good test-retest reliability, and good convergent and discriminant validity in clinical and community adult samples [14, 17]. There are only a few studies that have examined the psychometric properties of DERS-16 in clinical adolescent samples [12, 16]. These studies included samples consisting of individuals eating disorders and inpatients with severe mental illness, respectively, and not general outpatient child- and adolescent psychiatric (CAP) samples. Both studies primarily focus on factor analysis. Psychometric properties of DERS-16 for utility in transdiagnostic CAP-samples are thus lacking.

### ER, psychopathology, subgroups and gender differences

In a meta-analytic review, Cavicchioli [18] and colleagues propose that maladaptive ER is a significant predictor of psychopathology and that ER processes should be considered a transdiagnostic risk factor for psychopathology during development. Compas et al. [11] examined the relationship between internalizing and externalizing symptoms of psychopathology in childhood and adolescence, and different emotion regulation strategies. Overall, emotion regulation was significantly negatively associated with both internalizing and externalizing symptoms, whereas greater use of emotion regulation strategies was associated with lower levels of symptoms. Dysfunctional strategies for regulating emotions are often identified as underlying mechanisms in mental health [4], such as depression and anxiety [5], as well as different risk behaviors, such as nonsuicidal self-injury (NSSI) and substance abuse [6–8]. ER difficulties have also been shown to mediate the relationship between abuse and NSSI in a community sample of adolescents [19].

Earlier studies have explored different subtypes of ER difficulties based on DERS-36. Aleva and colleagues [20], for example, studied whether adolescents and young adults with borderline personality disorder had different patterns of ER difficulties, measured with the original DERS (36 items). They found three qualitatively different subgroups and argued that treatment should be tailored to fit different patterns of ER difficulties instead of a “one-size-fits-all” approach. Nordgren and colleagues [21] studied subgroups in adult eating disorder (ED) samples and identified three subgroups distinguishable on ED pathology and psychiatric comorbidity. The subgroup with more severe emotion dysregulation had higher levels of ED pathology and was more likely to engage in vomiting and binge eating, as well as substance abuse and self-harm. However, to our knowledge there are no studies that have examined subgroups of ER difficulties in a transdiagnostic CAP sample.

There also seem to be gender differences regarding which strategies adolescents use to regulate emotions. Two studies in adolescent community samples show that girls report higher levels of ER difficulties than boys, but also that girls report more frequent high-intense emotions and higher emotional instability than boys [22, 23]. Girls tend to report more social support-seeking and dysfunctional rumination, whereas boys report more passivity, avoidance, and suppression [24].

Taken together, the current study will fill some of the current knowledge gaps in the research field, in testing the psychometric properties in a clinical adolescent outpatient sample, in comparing ER difficulties in community and clinical adolescent samples and using a data-driven exploratory approach to identify groups of ER difficulties in the clinical sample. Comparing different ER groups on clinical variables can also improve our understanding of the role of ER difficulties in transdiagnostic CAP samples.

### Aims

The first part of the current study aimed at examining the psychometric qualities of DERS-16 in a CAP sample; whether the earlier underlying constructs of the bifactor model with five subscales could be replicated; examining criterion, convergent, and discriminant validity and whether DERS-16 was reliable over time.

In the second part of the study, we aimed at examine the clinical utility of DERS-16, describing and comparing difficulties in emotion regulation, as well as gender differences, in a clinical CAP sample and a community sample of adolescents. We further aimed to explore ER subtypes in the CAP sample using cluster analysis of the DERS-16 subscales and examining differences between subtypes on clinical variables, including risk-taking behavior, abuse experience, and psychiatric diagnoses.

For the first part we hypothesized that DERS-16 would show good psychometric properties in the clinical sample of adolescents. In the second part, we hypothesized that DERS-16 would distinguish between clinical and community adolescent samples with higher levels of ER difficulties in the CAP sample. For the ER subtypes in the clinical sample, we did not have a specific hypothesis, but wanted to exploratively investigate whether DERS-16 could be used for identifying different subgroups related to ER difficulties in the CAP sample that potentially could be of clinical relevance.

## Method

Self-report measures were administered in a community sample and a clinical sample of adolescents. DERS-16 was administered in a community sample of high-school students (*N* = 3,169), as a part of a large-scale study in schools. In the clinical sample (*N* = 281), DERS-16 was administered to adolescents enrolled at two outpatient child and adolescent psychiatric clinics.

### Procedure

#### Sample 1

Data were collected online in classrooms and during home studies in a representative sample of third year students in Swedish high schools during 2020–2021. The schools were selected based on information from the national school register and stratified to represent a normal population of third year Swedish high school students regarding school size and study program and were then randomized. If the principal of the school agreed, a date was set for data collection during lecture time. Specially appointed staff were present. Mandatory reporting was not possible since the survey was anonymous. To ensure the safety and wellbeing of the students, participants received written information where to turn for help and support, if needed.

Two hundred and ten schools with 7,752 students were selected and of these 110 schools with a total of 3,286 students completed the questionnaire. Four questionnaires were excluded because answers were obviously not meant seriously, resulting in 3,282 students and a response rate of 42.3%. Of the 3,282 participants, 113 were older than 19 years and were excluded in the present study, based on the United Nations definition of adolescence as the period between 13 and 19 years [25], which resulted in 3,169 adolescent participants in the present study. This study sample emanates from the survey “Young people, sex and the internet after #metoo” [26]. In the current study, only DERS-16 was used, together with demographic data.

#### Sample 2

Participants were recruited from two outpatient child and adolescent psychiatric (CAP) clinics during December 2020 to February 2022 in Östergötland county, Sweden. Consecutive sampling was used. Participants who were 15 years of age and above received written information about the study from the receptionist at the clinics. The questionnaires contained self-reported information on demographics, current psychiatric diagnosis, self-injury, trauma, substance use, ER, and related constructs (see below under measures). Participants answered the questionnaires anonymously in the waiting room and returned them to the receptionist in an envelope without any personal identifiable information. A total of 1,954 participants were eligible during the period. There is no accurate information available on how many of these were asked to participate, but of those eligible, 305 (15.6%) participants agreed to participate and began answering the questionnaires. Twenty-one participants had missing data on DERS-16 and were excluded, three participants were older than 19 years and were therefore excluded in the present study, resulting in 281 adolescents. Test-retest procedure for DERS-16 was conducted in a subsample (*n* = 37) with 14 to 21 days between the first and second administration. In the test-retest procedure, participants only answered the DERS-16 questionnaire and marked their questionnaires with a symbol so that the questionnaires from the two occasions could be paired together.

For both samples, participants received written information about the study and were informed that study participation was voluntary. The survey was anonymous. Participants were informed that that they gave informed consent to participate by answering the questionnaires, a procedure approved by the Swedish Ethical Review Authority (2019-05013-31, 2020–03611, 2020–06556), and with an advisory opinion (2020–03311).

### Participants

#### Sample 1

In the community sample, 3,169 adolescent high-school students aged 16–19 years (*M* = 18.1, *SD* = 0.5) were included. Of these, 1,383 (43.6%) were male, 1,761 (55.6%) were female and 25 (0.8%) identified as non-binary.

#### Sample 2

In the clinical CAP sample, 281 adolescents aged 15–19 years were included. Of the total sample, 206 participants reported their age (*M* = 16.2, *SD* = 1.0). Of the total sample, 55 (19.6%) were male, 218 (77.6%) were female and eight (2.8%) identified as non-binary. For participants’ demographics, see Table 1.

**Table 1:** Sociodemographic data for community (*N* = 3,169) sample and clinical (*N* = 281) sample of adolescents

		Community sample *n* (%)	Clinical sample *n* (%)
**Gender**			
	Boy	1383 (43.6)	55 (19.6)
	Girl	1761 (55.6)	218 (77.6)
	Non-binary identification	25 (0.8)	8 (2.8)
**Age** (*m*, *sd*)		18.12 (0.5)	16.17* (1.0)
**Parents’ occupation**			
	Fathers working	2768 (87.3)	249 (88.6)
	Mothers working	2798 (88.3)	216 (76.9)
**Parents’ education**			
	Fathers with university/college education	1250 (39.4)	106 (37.7)
	Mothers with university/college education	1738 (54.8)	149 (53.0)
**Country of origin**			
	Adolescents born in Sweden	2880 (90.9)	264 (94.0)
	Fathers born in Sweden	2573 (81.2)	252 (89.7)
	Mothers born in Sweden	2545 (80.3)	251 (89.3)
**Living situation**			
	With both parents	2009 (63.4)	155 (55.2)
	Alternating between both parents	362 (11.4)	48 (17.1)
	With one parent with or without new partner	630 (19.9)	55 (19.6)
	Alone or with siblings or partner	150 (4.7)	7 (2.5)
	In foster care or institution	18 (0.6)	11 (3.9)
Self-reported diagnoses**^,^ ^			
	Depression	-	112 (39.9)
	Anxiety disorder	-	79 (28.1)
	OCD	-	21 (7.5)
	ADHD/ADD	-	101 (35.9)
	Autism	-	59 (21.0)
	Eating disorder	-	69 (24.6)
	PTSD	-	23 (8.2)
	Other	-	14 (5.0)
	≥ 3 diagnoses***	-	58 (20.6)

Note. OCD = Obsessive compulsive disorder, ADHD/ADD = Attention deficit hyperactivity disorder/attention deficit disorder, PTSD = Post traumatic stress disorder. * *N* = 206, ** Each participant could report several diagnoses. ^ Self-reported diagnoses that participants had received from mental-health care. ***Any combination of the above listed diagnoses.

### Measures

#### Demographic information

Demographic questions were created for the purpose of the study assessing characteristics such as gender, age, parents’ occupation and education, own and parents’ immigrant background and living situation. Adolescents self-reported demographic information in fixed answer categories. Psychiatric diagnoses were self-reported in the clinical sample (sample 2) using the item: “Which diagnosis/es have you received at the child- and adolescent clinic”, with fixed response categories for the most common disorders (Table 1).

#### Nonsuicidal self-injury and risk behaviors

In sample 2, life-time prevalence of NSSI was assessed with the NSSI-item from the Self-Injurious Thoughts and Behaviors Interview [27], short-form and self-report version. Experience of drug and alcohol use was assessed with two questions developed for the purpose of this study with fixed answer categories of “yes” and “no”.

#### Abuse experience

Three items measuring physical, sexual and emotional abuse with response alternatives “yes” or “no” were derived from the Linköping Youth Life Experience Scale [28] and used to measure lifetime prevalence of abuse experience in sample 2.

#### Emotion regulation and related constructs

*DERS-16* [14] was administrated in sample 1 and sample 2. DERS-16 measures difficulties in ER and consists of 16 items rated on a five-point Likert scale from “almost never” to “almost always”, the score ranges from 16 to 80. Higher scores indicate higher levels of difficulties. DERS-16 has shown good psychometric properties [14]. In some studies, removing items 14 and 16 have resulted in better model fit when conducting factor analysis [15, 29]. In the present study, Cronbach’s alpha was 0.95 for the total scale, 0.87 for non-acceptance, 0.90 for impulse, 0.89 for goals, 0.89 for strategies and 0.83 for clarity, in sample 1, indicating good to excellent internal consistency. In sample 2 Cronbach’s alpha was 0.92 for the total scale. For more information on sample 2, see results.

*Emotion Regulation Questionnaire* (ERQ; [30]) measures two different ER strategies, cognitive reappraisal and expressive suppression. High scores on Cognitive reappraisal indicate higher ER and higher scores on expressive suppression indicate lower ER. The measure contains 10 items rated on a seven-point Likert scale, six for cognitive reappraisal and four items for expressive suppression, score ranges from 6 to 42 on cognitive reappraisal scale, and from 4 to 28 on expressive suppression scale. Cronbach’s alpha for sample 2 in the current study was 0.70 for the expressive suppression scale and 0.82 for the cognitive reappraisal scale, indicating acceptable and good internal consistency, respectively.

*Toronto Alexithymia Scale* (TAS-20; [31, 32]) measures alexithymia and has 20 items, ranging from totally right to totally wrong on a five-grade Likert scale, the score ranges from 20 to 100. The questionnaire comprises three subscales: difficulties identifying emotions (Identifying); difficulties describing emotions (Describing) and difficulties externalizing emotions (External focus). Higher scores indicate higher levels of alexithymia. TAS-20 is one of the most used self-report scales for alexithymia [33] and has shown good reliability and validity [34, 35]. In this study, Cronbach’s alpha was 0.83, indicating good internal consistency in sample 2.

### Statistical analysis

Statistical analyses were performed using the SPSS v. 29.0 software package (SPSS Inc, Chicago, IL), and the lavaan library for the R statistical software package version 4.0.3 [36, 37]. Descriptive statistics were analyzed using frequencies and percentages, mean values, and standard deviations, with cross-tabulation using chi square (χ2) and independent sample t-test for comparing differences between groups. Effect sizes (Cohen’s *d*) were calculated using Cohen’s [38] criteria of 0.20, 0.50 and 0.80 for small, medium and large effect. For effect sizes in cross-tabulation chi-square test, Cramer’s V was used, using Cohen’s [38] criteria of 0.10, 0.30, 0.50 for small, medium, and large effect. Intraclass correlation coefficients were computed to determine the test-retest reliability of the DERS-16. Internal consistency was assessed using Cronbach’s alpha (α). The questionnaires of related constructs aimed at exploring convergent and discriminant validity. Pearson’s product-moment correlations were conducted to evaluate associations between the DERS variables and other relevant self-report measures for testing convergent and discriminant validity.

In order to investigate the underlying factor structure of the DERS-16 using the present sample, confirmatory factor analysis was carried out using the lavaan library for the R statistical software package version 4.0.3 [36, 37]. Models were fitted using the Robust Maximum Likelihood estimator (MLR) and evaluated according to the widely accepted cut-off criteria by Hu and Bentler [39], considering robust fit measures where available. Based on results from earlier research evaluating the factor structure of DERS-16 in different samples [12, 29, 40] three models were fitted and evaluated: a correlated-factors model, a higher-order model and a bifactor model with five group factors, where a general ER factor was specified as independent and orthogonal relative to the five uncorrelated “group” factors. As the Clarity subscale had only two items, factor loadings were constrained to 1. Models were compared using Akaike information criterion (AIC) and Bayes information criterion (BIC).

Two-step cluster analysis with log-likelihood distance measures was used in SPSS as an explorative tool to reveal natural cluster clusters within the dataset for detection of potentially different groups of difficulties in ER. The subscales of DERS-16 were selected as cluster variables and automatic selection of clusters with a maximum of 15 were used. The variables were standardized before clustering.

Chi-square (χ^2^) analysis and one-way analysis of variance (ANOVA) were conducted to test differences between the clusters, where an effect size of 0.01, 0.06 and 0.14 for Eta Squared indicated a small, medium and large effect, respectively [38]. Tukey HSD test was used to control for multiple comparisons with the continuous data.

## Results

### Validation of DERS-16 in a clinical sample of adolescents

#### Validity

Confirmatory factor analysis initially yielded a poor model fit for both the correlated-factors model and the higher-order model (respectively, AIC = 12652.49, BIC = 12801.67, RMSEA = 0.084, CFI = 0.931, TLI = 0.913, SRMR = 0.061; and AIC = 12687.98, BIC = 12818.96, RMSEA = 0.090, CFI = 0.916, TLI = 0.900, SRMR = 0.073). For the bifactor model, the maximum likelihood function did not converge. Having examined modification indices, it was decided to respecify the models and fit them again. This resulted in three new, reduced models without DERS-16 items 14 and 16. The same modification was previously carried out by Westerlund and Santtila [29]. Model fit for the reduced correlated-factors model as well as the higher-order model was approaching acceptability according to the prespecified criteria (respectively, AIC = 11026.61, BIC = 11161.23, RMSEA = 0.068, CFI = 0.961, TLI = 0.948, SRMR = 0.050 and AIC = 11043.52, BIC = 11163.59, RMSEA = 0.073, CFI = 0.953, TLI = 0.940, SRMR = 0.060) and the reduced bifactor model with five subscales was found to be within the limits for relatively good fit (AIC = 11003.85, BIC = 11153.02, RMSEA = 0.059, CFI = 0.973, TLI = 0.960, SRMR = 0.051). The reduced bifactor model also had the lowest AIC and BIC indices.

For total DERS-16 score, there was a significant large positive correlation (*r* =.52, *p* <.01). with TAS-20 total score, which measuring alexithymia. The subscale in TAS-20 measuring difficulties identifying feelings had significant medium to large correlations with all the DERS-16 subscales (*r* =.41– 0.65, *p* <.01). The ERQ scale expressive suppression (*r* =.27, *p* <.01) had a small but significant correlation with DERS-16 total score, and small significant correlations with all subscales in DERS-16, except the impulse scale (Table 2). DERS-16 thus correlated significantly with other similar constructs, indicating good convergent validity.

**Table 2:** Pearsons’s product correlations between DERS-16, total and subscales, and other self-reported measures

		TAS-20			ERQ	
DERS-16	TAS-20 Total	Difficulties describing feelings	Difficulties identifying feelings	Externally orientated thinking	Cognitive reappraisal	Expressive suppression
DERS-16 Total	0.52**	0.43**	0.63**	*ns*	− 0.18**	0.27**
Clarity	0.63**	0.58**	0.65**	0.16*	− 0.20**	0.28**
Goals	0.35**	0.32**	0.41**	*ns*	− 0.20**	0.15*
Impulse	0.44**	0.33**	0.46**	0.19**	− 0.14*	*ns*
Strategies	0.38**	0.31**	0.54**	*ns*	− 0.17**	0.27**
Nonacceptance	0.32**	0.27**	0.48**	*ns*	*ns*	0.28**

NOTE. DERS-16 = Difficulties with emotion regulation scale, 16-item version, CLARITY = Lack of Emotional Clarity; GOALS = Difficulties Engaging in Goal-Directed Behavior; IMPULSE = Impulse Control Difficulties; STRATEGIES = Limited Access to Effective Emotion Regulation Strategies; NONACCEPTANCE = Nonacceptance of Emotional Responses, TAS-20 = Toronto Alexithymia Scale, ERQ = Emotion Regulation Questionnaire. *Correlation is significant at the 0.05 level (2-tailed). **Correlation is significant at the 0.01 level (2-tailed).

For discriminant validity the correlations with the cognitive reappraisal scale in ERQ were explored. DERS-16 total scale had significant small correlation with the subscale cognitive reappraisal (*r* = −.18, *p* <.01) in the ERQ, and all the subscales of DERS-16 had small but significant correlations with the cognitive reappraisal scale (*r* = −.14– − 0.20, *p* <.05), except for the non-acceptance scale. Taken together this indicated good discriminant validity (See Table 2).

There were significant differences between mean average scores on both the total DERS-16 score and the subscales nonacceptance, impulse, goals, strategies and clarity between the community sample and clinical sample (*p* <.001) for all comparisons with medium to large effect sizes (Table 3) supporting criterion validity. The clinical groups reported higher overall scores indicating that the DERS-16 differentiated a clinical from a community adolescent population.

**Table 3:** Emotion regulation difficulties in a community (*N* = 3,169) and clinical adolescent sample (*N* = 284), means, standard deviations

	Community sample (*N* = 3,169)				Clinical sample (*N* = 281)				Statistics					
	Total	Boys (*n* = 1,383)	Girls (*n* = 1,761)	Non-binary identification (*n* = 25)	Total	Boys (*n* = 55)	Girls (*n* = 218)	Non-binary identification (*n* = 8)	Total community vs. clinical sample		Boy vs. Girl community sample*		Boy vs. Girl clinical sample*	
DERS 16	*M (SD)*	*M (SD)*	*M (SD)*	*M (SD)*	*M (SD)*	*M (SD)*	*M (SD)*	*M (SD)*	*p*	*d*	*p*	*d*	*p*	*d*
Total	36.54 (15.74)	31.33 (13.83)	40.45 (15.90)	49.76 (17.41)	50.57 (14.32)	42.85 (14.90)	52.50 (13.56)	51.00 (14.16)	< 0.001	0.90	< 0.001	0.61	< 0.001	0.70
Clarity	4.42 (2.10)	3.94 (1.97)	4.78 (2.11)	5.92 (2.77)	5.78 (2.24)	4.95 (2.16)	5.97 (2.21)	6.38 (2.26)	< 0.001	0.64	< 0.001	0.41	0.001	0.46
Goals	8.45 (3.65)	7.22 (3.43)	9.38 (3.52)	10.76 (3.62)	11.41 (3.09)	10.18 (3.69)	11.70 (2.86)	11.88 (2.85)	< 0.001	0.82	< 0.001	0.62	0.003	0.50
Impulse	5.90 (3.27)	5.18 (2.82)	6.45 (3.47)	7.64 (3.80)	8.66 (3.73)	8.16 (3.53)	8.34 (3.80)	7.38 (2.92)	< 0.001	0.83	< 0.001	0.40	*ns*	
Strategies	10.79 (5.49)	9.09 (4.73)	12.05 (5.67)	15.80 (5.47)	15.54 (5.36)	12.65 (5.48)	16.27 (5.08)	15.75 (5.82)	< 0.001	0.87	< 0.001	0.56	< 0.001	0.70
Non-acceptance	6.98 (3.62)	5.90 (3.26)	7.78 (3.66)	9.64 (4.21)	9.18 (3.64)	6.91 (3.51)	9.73 (3.46)	9.63 (3.40)	< 0.001	0.61	< 0.001	0.54	< 0.001	0.82

Note. DERS-16 = Difficulties with emotion regulation scale, 16-item version. * Due to small number, non-binary identification was not included in analysis. Between group comparisons with independent sample t-test and effect sizes (Cohen’s d).

#### Reliability

DERS-16 were administered to 37 participants on two occasions with two to three weeks apart. Intraclass correlation coefficients were 0.91 (*p* <.001) for the total scale, 0.81 (*p* <.001) for non-acceptance, 0.86 (*p* <.001) for goals, 0.86 (*p* <.001) for impulse, 0.92 (*p* <.001) for strategies and 0.63 (*p* =.002) for clarity. Results indicate good to excellent test-retest reliability for the total and the subscales (except the clarity-scale) in the current clinical sample of adolescents. Internal consistency coefficients (Cronbach’s α) were 0.92 for the total scale, 0.84 for non-acceptance, 0.90 for impulse, 0.85 for goals, 0.84 for strategies and 0.81 for clarity, indicating very good to excellent internal consistency.

### Difficulties with emotion regulation in a clinical and community sample of adolescents

Mean level of total DERS-16 score in the community sample was 36.54 (*SD* = 15.74) compared to *M* = 50.57 (*SD* = 14.32) in the clinical sample. Independent samples *t*-test showed that the clinical sample had significantly higher scores than the community sample, both in the total scale *t*(343) = 15.61, *p* <.001, Cohen’s *d* = 0.90, and for all the subscales (*p* <.001) with *d’s* ranging between 0.61 and 0.90, indicating medium to large effects (Table 3), with higher levels of difficulties with ER in the clinical sample.

### Gender differences in difficulties with emotion regulation

In the clinical sample, a major proportion were girls (77.6%) compared to the community sample (55.6%). In both samples, girls reported significant higher scores than boys on DERS-16, on the total scale *t*(3142) = 16.89, *p* <.001, Cohen’s *d* = 0.61 for community sample and *t*(271) = 4.62, *p* <.001, Cohen’s *d* = 0.70 for the clinical sample and on all the subscales (*p* <.001) in the community sample, and in the clinical sample (*p* =.003 to *p* <.001), except for subscale Impulse (ns). In the clinical sample there was, however, no significant difference between boys and girls on the impulse subscale. (Table 3). Boys in the community sample (*n* = 1,383) reported a total mean score of 31.33 (*SD* = 13.83) on the DERS-16, and girls (*n* = 1,761) reported a score of *M* = 40.45 (*SD* = 15.90). In the clinical sample, boys (*n* = 55) reported a total mean score of 42.85 (*SD* = 14.90), and girls (*n* = 218) reported *M* = 52.50 (*SD* = 13.56). Adolescents who identified as non-binary (*n* = 25) in the community sample reported the highest levels of ER difficulties (*M* = 49.76, *SD* = 17.41) in that sample. Levels of ER difficulties for adolescents who identified as non-binary were also high (*M* = 51.00, *SD* = 14.16) in the clinical sample (*n* = 8). No statistical analysis was performed, however, due to small sub-sample sizes.

### Two-Step cluster analysis of DERS-16

A two-step cluster analysis using the five subscales in DERS-16 as cluster variables to explore subtypes of difficulties with ER was conducted. The cluster analysis resulted in three quantitatively differentiated clusters, with a silhouette coefficient of 0.4 indicating a fair cluster homogeneity, and a size ratio between clusters of < 3. The three-cluster solution showed no qualitative differences with variation in the DERS-16 subscales. The subscale profile was the same for all three clusters, ranging from lower to higher levels of ER difficulties in the Minor, Moderate and Severe clusters. Based on mean scores of the DERS-16 subscales in the clusters, Cluster 1 (*n* = 65, 23.1%) was labelled Minor (ER difficulties), Cluster 2 (*n* = 160, 56.9%) was labelled Moderate (ER difficulties) and Cluster 3 (*n* = 56, 19.9%) was labelled Severe (ER difficulties). The subscale “strategies”, i.e., limited access to effective emotion regulation strategies, had the highest predictor importance, followed by the subscales: goals, clarity, impulse, and non-acceptance.

#### Cluster profile analysis

Chi-square test and one-way ANOVAs were executed to examine differences between the three clusters regarding experience of risk-taking behaviors (NSSI, drugs, and alcohol), abuse (emotional, physical, and sexual), age, and self-reported psychiatric diagnoses (Table 4). Significant differences between the three clusters regarding gender were confirmed, (χ^2^ (2, *N* = 273) = 17.18, *p* <.001). More girls were represented in the Severe and Moderate ER difficulties clusters (89.3% and 80.0%) than in the Minor cluster (61.5%), and the proportion of boys was larger in the Minor cluster (36.9%) than in the Moderate and Severe (16.3% and 8.9%, respectively). ES were small to medium.

**Table 4:** Comparisons of demographics, risk-taking behavior, psychiatric diagnoses, abuse experience and personality dimensions for three clusters of minor (*n* = 66), moderate (*n* = 162) and severe (*n* = 56) ER difficulties, frequencies, percentages, mean values, standard deviations

	Minor *n* = 64–65*	Moderate *n* = 157–160*	Severe *n* = 53–56*	Statistics	
	*n* (%)	*n* (%)	*n* (%)	*P*	ES
**Demographics**					
Boy	24 (36.9)	26 (16.3)	5 (8.9)	< 0.001	0.25
Girl	40 (61.5)	128 (80.0)	50 (89.3)	< 0.001	0.23
Non-binary identification	1 (1.5)	6 (3.8)	1 (1.8)	**	
Age M (SD)	16.16 (1.01)	16.22 (1.00)	16.05 (1.00)	*ns*	
**Risk-taking behavior**					
NSSI	17 (26.2)	105 (66.5)	49 (89.1)	< 0.001	0.44
Alcohol	31 (47.7)	83 (52.2)	33 (60.0)	*ns*	
Drugs	7 (10.8)	20 (12.6)	15 (27.3)	0.017	0.17
Self-reported diagnoses***					
Depression	11 (16.9)	70 (43.8)	31 (55.4)	< 0.001	0.27
Anxiety disorders	8 (12.3)	50 (31.3)	21 (37.5)	0.004	0.20
OCD	5 (7.7)	8 (5.0)	8 (14.3)	**	
ADD/ADHD	26 (40.0)	51 (31.9)	24 (42.9)	*ns*	
Autism	12 (18.5)	36 (22.5)	11 (19.6)	*ns*	
PTSD	1 (1.5)	14 (8.8)	8 (14.5)	**	
Eating disorder	19 (29.2)	40 (25.0)	10 (17.9)	*ns*	
≥ 3 diagnoses	5 (7.7)	35 (21.9)	18 (32.7)	0.003	0.20
**Abuse**					
Emotional	21 (32.3)	106 (67.5)	47 (85.5)	< 0.001	0.38
Physical	9 (13.8)	50 (31.8)	26 (47.3)	< 0.001	0.24
Sexual	5 (7.8)	38 (24.2)	29 (54.7)	< 0.001	0.35
**ER difficulties**					
DERS-16 total scale M (SD)	31.35 (6.81)	51.40 (6.52)	70.50 (4.85)	< 0.001	0.81

Note. NSSI = Nonsuicidal self-injury, OCD = Obsessive compulsive disorder, ADHD/ADD = Attention deficit hyperactivity disorder/attention deficit disorder, PTSD = Post traumatic stress disorder. *Number varies due to missing data on some variables. **Not applicable due to small number. *** Self-reported diagnosis from the child- and adolescent services. Chi-square for group comparisons for categorical data and one-way ANOVA for comparisons for continuous data, effect sizes (Cramer’s V and Eta-squared).

Regarding risk-taking behaviors, the three clusters were significantly differentiated on lifetime prevalence of NSSI (χ^2^(2, *N* = 278) = 53.63, *p* <.001) and drug use (χ^2^(2, *N* = 279) = 8.12, *p* =.02), with more adolescents having these experiences in the Severe cluster of ER difficulties (89.1% and 27.3%), while fewer adolescents reported NSSI or drug use in the Minor cluster (26.2% and 10.8%). ES for NSSI was medium to large and small for drugs. There was no significant difference between the three clusters for consumption of alcohol. There were significant differences between the three cluster for depression (χ^2^(2, *N* = 281) = 20.87, *p* <.001) and anxiety disorders (χ^2^(2, *N* = 281) = 11.26, *p* =.004). More adolescents in the Severe cluster reported having received a diagnosis of depression (55.4%) or anxiety disorders (37.5%), than in the Minor cluster (16.9% and 12.3%). ES were small to medium. There were no significant differences for the other diagnoses. For some diagnoses, however, the groups were too small to run between group analysis. To examine differences between clusters on psychiatric co-morbidity, a variable was created with three or more diagnoses, which was significantly different between the clusters (χ^2^(2, *N* = 280) = 11.66, *p* =.003), small to medium effect). There were more adolescents who had received three or more diagnoses (32.7%) in the Severe cluster than in the Minor cluster (7.7%). Furthermore, there were significant differences between the clusters on self-reported experience of emotional (*χ*^2^(2, *N* = 277) = 39.45, *p* <.001), physical (*χ*^2^(2, *N* = 277) = 15.88, *p* <.001), and sexual (*χ*^2^(2, *N* = 274) = 33.74, *p* <.001) abuse, with higher frequencies of abuse experiences in ascending order from Minor to Moderate to the most Severe cluster. Es were small to medium and medium sized. There were significant differences (F(2,130.4) = 584.2, *p* <.001) in mean scores of DERS-16 total scale for the three clusters, *M* = 31.35 (*SD* = 6.81) for the Minor cluster, *M* = 51.4 (*SD* = 6.52) for the Moderate cluster and *M* = 70.5 (*SD* = 4.85) for the Severe cluster, with a large effect size. Tukey HSD indicated that all three clusters differed significantly (*p* <.001) in mean scores at the total DERS-16 scale.

## Discussion

DERS-16 showed satisfactory psychometric qualities in this CAP sample. DERS-16 successfully distinguished adolescents in the clinical sample from adolescents in the community sample. Furthermore, a two-step cluster analysis identified three clusters: Minor, Moderate and Severe ER difficulties. Adolescents with highest level of self-reported ER difficulties reported significantly more risk behaviors such as NSSI and drug use, depression and anxiety, and exposure to abuse, and higher levels of comorbidity.

### Psychometric properties of DERS-16

Earlier research has studied DERS-16 mainly in adult populations, both in clinical and community samples [14]. To investigate the underlying construct of DERS-16 in this CAP sample, a confirmatory factor analysis was conducted, and was partly unsuccessful, likely due to the small sample size given the complexity of the models evaluated. The fact that more acceptable model fit was the result of reducing the models by two items draws attention to items 14 and 16 and where they best belong. Items 14 and 16 have been debated in earlier research and have been deleted due to cross loadings [16, 44]. When, following respecification, the reduced models were evaluated, the bifactor model turned out to be the one fitting best to sample data while the others were found to be approaching acceptable fit. The interpretation of this is that confirmatory factor analysis using the present sample cannot conclusively single out one of the models as superior, but rather weakly supporting all three models. These results support the fact that DERS-16 in the current CAP sample can be considered to measure a multidimensional phenomenon consisting of one general factor and other specific factors. DERS-16 has shown to have a poor fit as a unidimensional model [16]. In the current study, results from the total scale are reported to enable comparisons with other studies that present DERS-16 total scale scores. The subscales were also used in the analysis, based on the CFA that showed that the subscales do in fact measure different factors of ER that are clinically relevant.

The convergent and discriminant validity was good, DERS-16 correlated positively with related constructs, measures of alexithymia and expressive suppression, and negatively with hedonic capacity. The positive correlations with the difficulties describing and identifying feelings subscales from TAS-20 correspond well with Gratz and Roemer’s [3] conceptualization of ER where awareness and understanding of emotions are highlighted. The internal consistency and test-retest reliability were good to excellent. Overall, the DERS-16 was shown to have satisfactory psychometric properties in our clinical adolescent sample.

### ER differences in a clinical and a community sample of adolescents

The mean score of DERS-16 (*M* = 36.54) in the adolescent community sample presented here (mean age 18.12 years) was in line with previous studies using community samples, including Bjureberg et al. (2016; *M* = 42.90 and *M* = 33.57, respectively) although these were adult samples (mean age 24.68 and 21.75, respectively). There is currently a lack of comparative data on DERS-16 for adolescents in both community and clinical samples, except for Monell et al. [12] data on DERS-16 in an adolescent eating disorder sample. In this study, DERS differentiated adolescents in the clinical sample from adolescents in the community sample, with significantly higher levels of self-reported difficulties with emotion regulation for the clinical sample. The mean values of ER difficulties in the CAP sample *M* = 50.57 (*SD* = 14.32) in our study can be compared to Monell’s (2022) data on a clinical eating disorder sample, *M* = 46.08 (*SD* = 14.85). To our knowledge, no other studies have presented mean values on adolescent community samples or adolescent general CAP samples. The mean age in the community sample was higher than in the CAP sample in the current study (mean age 18.12 and 16.17 years, respectively). Scores on the original DERS (36-items), tended to decrease with age [41], which could suggest that the difference between the clinical and community samples in the current study could have been smaller if the samples had been the same age. Even though age could influence ER, partly due to brain maturity and increased frontal functioning that comes with increased age [42], participants in our community sample were still adolescents with ongoing brain development and it is not likely that the large difference noted here is solely attributed to a somewhat larger mean age in the community sample.

### Gender differences in self-reported ER difficulties

Girls were overrepresented in both samples in the current study, (55.6% respectively 77.6%). In the present study, girls reported significantly higher levels of ER difficulties than boys, *M* = 52.50 (*SD* = 13.56) and *M* = 42.85 (*SD* = 14.90), respectively. In both the community and the CAP sample in the current study, girls reported higher levels of ER difficulties than boys in the total scale and all subscales, except in the impulse-scale in the CAP sample. It is not clear whether these differences are due to actual gender differences in difficulties in regulating emotions or whether it has to do with gender differences in response style. In previous research there is some support for girls being more hyperresponsive in self-report questionnaires, whereas boys tend to underreport [43]. To our knowledge, there are no previous data on gender differences on DERS-16 in adolescent samples. In adult samples, however, there are no, or small, gender differences in reports of ER difficulties using DERS-16 [40, 44]. When considering ER difficulties in general, there is some evidence that adolescent girls tend to report more difficulties [22, 23]. Gender differences have also been found in emotion dimensions that are connected to ER, such as emotion frequency, intensity and instability [45], where girls report more frequent high-intense emotions, and higher emotional instability than boys [45]. Boys report less emotion differentiation than girls, and high differentiation is associated with more effective regulation of emotions [46]. Adolescents identifying as non-binary were too few to analyze further, but the mean value of DERS-16 indicates that this group experiences severe difficulties in emotion regulation.

### ER subtypes in a CAP sample

Cluster analysis of the DERS-16 sub-scales in the CAP-sample resulted in three clusters named Minor, Moderate and Severe ER difficulties. The aim was to explore if there were different groups of ER difficulties in the CAP sample and examine whether clinically relevant characteristics differed between these groups. The group that reported Moderate ER difficulties was the largest (56.9%), followed by those with least difficulties (23.1%), and the group with the highest self-reported ER difficulties (19.9%). The three clusters that were identified did not differ qualitatively with variation in the DERS-16 subscales, but rather represented quantitatively different severity dimensions, ranging from low to high of ER difficulties. There thus was no advantage with the clusters in the current sample as compared to a dimensional approach of ER difficulties. This contrasts with the results of Aleva and colleagues [20] who found qualitatively different subgroups of emotion regulation patterns when conducting a latent profile analysis of the original form of DERS, 36 items, in young people with borderline personality disorder. Adolescents in the cluster with the highest DERS-16 scores in the current CAP sample, had considerably higher mean scores (*M* = 70.50, *SD* = 4.85) than an adult clinical sample with NSSI (*M* = 57, *SD* = 13.05) that met at least three diagnostic criteria for borderline personality disorder [14]. The adolescents in the Severe cluster furthermore had more depression and anxiety diagnoses, as well as more comorbidity in psychiatric diagnoses. These findings are in line with earlier research that implicates that difficulties in ER are associated with overall psychopathology and specific diagnoses, such as depression and anxiety [7]. Due to the relatively small sample, it is not possible to conclude any associations with other psychiatric diagnoses. Adolescents in the Severe cluster also reported more NSSI and drug use. It is well known that there is an association between NSSI and ER and earlier research shows that ER is a core mechanism in the development and maintenance of NSSI [19, 47]. The relationship between ER difficulties and addiction in adults and adolescents is also well-studied [48–50]. There is also support for a reciprocal relationship between posttraumatic stress syndrome (PTSD) and ER [51]. In our data the differences in PTSD diagnoses were not significant between clusters, however adolescents in the cluster with highest scores on DERS-16 reported significantly (*p* <.001) higher levels of all three types of abuse.

ER difficulties are generally associated with mental health problems [7, 52] and are identified as a transdiagnostic risk factor for psychopathology during development [18]. Interestingly, the adolescents reporting lowest scores on DERS-16 ER difficulties, had a mean value of ER difficulties on DERS-16 (*M* = 31.35, *SD* = 6.81) in line with an adult community sample (*M* = 33.57, *SD* = 13.14) in Bjureberg et al. [14]. In this group with low self-reported ER difficulties, there was a significantly larger proportion of boys (36.9%) than in the Moderate and Severe clusters (16.3% and 8.9%, respectively) and a larger proportion of self-reported eating disorders (29.2%) than in the Moderate (25.0%) and Severe (17.9%) clusters. The adolescents in the Minor cluster also had significantly lower levels of comorbidity. The difference in eating disorder prevalence was not significant, however, and the participants did not specify which type of eating disorder they had.

### Strengths and limitations

This study suggests that DERS-16 can be a useful and clinically relevant measure to distinguish clinical from community adolescent samples and to identify relevant subgroups in transdiagnostic CAP samples. However, the result should be interpreted with caution due to the limitations of the study. The study is based on cross-sectional data and therefore no causal relationships can be presented, the results indicate that there are associations between ER difficulties and psychopathology, risk behaviors and abuse but no assumptions about causality can be made. For example, there could potentially be an increased likelihood of engaging in risk behaviors if someone has greater difficulties in ER, or vice versa.

There are differences in the clinical and community samples that could possibly have influenced the result, such as age and gender. Another limitation is that only self-report measures were used, and results can therefore be influenced by shared method variance, both in the way that data is reported but also that some of the different measures have related constructs. The lack of information about the dropouts hampers generalization of the results. Yet another limitation is that the clusters identified in the study did not differ qualitatively with variation in the DERS-16 subscales, but rather identified groups that differ in severity. Despite this weakness of the analysis method, the results of the cluster analysis were interesting in relation to psychopathology and risk behaviors and helped answer the question regarding clinically relevant subtypes in the CAP-sample.

On the other hand, the strength of the study is the relatively large community sample. Multiple comparisons always pose a risk for Type 1 error, which also had to be taken in account when interpreting results.

### Clinical implications

Overall, the DERS-16 was found to have satisfactory psychometric qualities and seems to work well in a clinical CAP sample. It also can distinguish clinical from community samples in adolescents. For adolescents, measurements need to be brief and easy to administer wherefore this short form for measuring ER difficulties is clinically useful.

As adolescents with high levels of ER difficulties seem to be a more burdened group on several clinical variables, it is especially important to identify these individuals. Measuring ER difficulties in a reliable and valid manner could be one potential way to do so. Interestingly, a group of adolescents with low levels of ER difficulties that were less burdened on clinical variables was also identified in the CAP sample. This highlights the importance of targeting ER difficulties in the treatment of adolescents in CAP services, as well as identifying those who would benefit the most from such interventions.

## Data Availability

The datasets presented in this article are not readily available because the dataset is not public. Requests to access the datasets should be directed to kristina.holmqvist.larsson@liu.se or carl-goran.svedin@mchs.se.
